# Identifying Demographic Factors Affecting the ECG Duration Collected Using a Single‐Lead ECG Patch Device

**DOI:** 10.1111/anec.70068

**Published:** 2025-05-04

**Authors:** Dillon J. Dzikowicz, Mehmed Aktas, Betty Mykins, Xiaojuan Xia, Wojciech Zareba, Jean‐Phillippe Couderc

**Affiliations:** ^1^ School of Nursing University of Rochester Rochester New York USA; ^2^ Clinical Cardiovascular Research Center University of Rochester Medical Center Rochester New York USA

**Keywords:** diagnostic techniques and procedures, electrocardiography ambulatory, heart function tests, monitoring ambulatory

## Abstract

**Introduction:**

Atrial fibrillation (AF), affecting 3% of the US adults, is the most common arrhythmia. While ambulatory electrocardiogram (ECG) monitoring is essential for AF detection, conventional technologies have diagnostic limitations due to AF's sporadic nature. ECG patches offer extended monitoring periods, though their effectiveness is primarily limited by deteriorating skin‐electrode contact rather than battery or memory constraints.

**Objectives:**

This analysis reports our experience with the Zio ECG patch (iRhythm, San Francisco, CA) in 256 AF patients.

**Method:**

We analyzed human and technical factors and their association with ECG recording duration using previously recorded data which employed the ECG patch as a reference. Descriptive statistics and logistic regression were used to identify associations.

**Results:**

Body mass index (BMI) was found to be an independent predictor of poorer compliance in a dose‐dependent manner (*B* = −0.046, OR = 0.955, 95% CI: 0.916–0.996, *p* = 0.033). Loss of adhesive was the primary reason for poor compliance (*n* = 25; 11%). These findings can guide researchers and clinicians in determining the appropriateness of a 14‐day ECG patch based on expected wear time and patient compliance.

**Conclusion:**

BMI significantly impacts ECG patch compliance, primarily through adhesive failures. These findings indicate the need for improved adhesive technologies for higher BMI patients. Future device development should prioritize maintaining electrode‐skin contact across diverse patient populations.

**Trial Registration:**

ClinicalTrials.gov Identifier: NCT04267133

## Graphical abstract

Body mass index (BMI) significantly impacts electrocardiogram (ECG) patch compliance, with higher BMI associated with reduced wear time. Patients with obesity experience higher rates of adhesive failure, limiting effective 14‐day monitoring. These findings highlight the need for improved adhesive technologies to optimize ambulatory ECG monitoring.

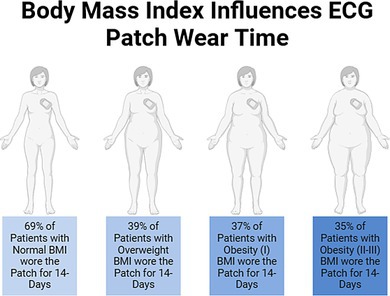

## 1 Introduction

Atrial fibrillation (AF) is the most common arrhythmia in the United States, affecting 3% of adults. The diagnosis of AF presents unique challenges due to its often asymptomatic nature and unpredictable presentation (Williams et al. 2020; January et al. 2019). While a resting 12‐lead electrocardiogram (ECG) remains the diagnostic gold standard, its yield is limited to < 1% of new AF cases (Nagata et al. 2020), primarily due to the transient nature of the arrhythmia and restricted availability of ECG testing. This limitation has driven the development of longer‐term ambulatory monitoring solutions.

To address these diagnostic challenges, ECG patch devices have emerged as a significant advancement in continuous cardiac monitoring. These devices integrate a sensor system, microelectronic circuitry for recording and storage, and an internal battery within a flexible synthetic unit, allowing for extended monitoring periods beyond traditional methods. While technical capabilities such as battery life and memory capacity have been optimized, maintaining consistent electrode‐skin contact over extended periods remains the primary challenge. Recent studies suggest that median wear times for ECG patches are closer to 11–12 days, which is notably shorter than the manufacturer‐reported compliance of 14 days. However, these studies do not account for factors such as body mass index (BMI), which may significantly influence adherence and device performance (Bouzid et al. 2022).

The success of long‐term ECG monitoring heavily depends on patient compliance, as these devices are typically worn for up to 14 days. Although patients generally report better tolerance of ECG patches than traditional Holter monitors, skin irritation and adhesive failure may still occur and can limit wear time. While prior research has focused on ECG signal quality, little to no papers have focused on the factors that influence device wear time and overall device compliance (Campero Jurado et al. 2023). Understanding compliance factors is especially important as AF management increasingly focuses on measuring AF burden, which requires consistent, long‐term monitoring (Varma et al. 2021; January et al. 2019).

Building on these observations, the present study aimed to identify demographic and clinical predictors of ECG patch compliance. We analyzing existing data from patients post‐ablation and post‐cardioversion providing valuable insights into understanding monitoring duration and overall adherence for long‐term ECG patch use.

## 2 Methods

### 2.1 Study Design

This secondary analysis of the Home‐based Videoplethysmographic Detection of Atrial Fibrillation Study (6/2017‐6/2021; R01HL137617) recruited patients with paroxysmal or persistent AF undergoing catheter ablation or trans‐thoracic electrical cardioversion to test novel videoplethysmographic software to detect incidental AF compared to the standard ECG. Inclusion criteria included adults ≥ 18 years of age with medically managed symptomatic paroxysmal or paroxysmal AF and successful return to sinus rhythm after catheter ablation or electrical cardioversion. Exclusion criteria included patients with implanted cardiac devices (pacemaker, cardiac resynchronization therapy, implantable cardioverter defibrillator) and a ventricular pacing requirement ≥ 70%, those without internet access, known allergic reactions to adhesives or hydrogels or with a family history of adhesive skin allergies, those unable to cooperate with the protocol, and those with an inability to use the technology (e.g., blind, tremor, Parkinson Disease, etc.).

### 2.2 Patient Demographics

For demographics, a subject's age (years), race (White, Black/African American, Asian, Indian/Alaska Native, Native Hawaiian or Pacific Islander, more than 1 Race, Unknown), weight (kilograms), height (meters), and BMI (kg/m^2^) based on the electronic medical record were recorded. During the consent process, research staff members asked the subject about work status (Do you still work?; Full‐time, Part‐time, Not Currently Working), job activity (If you are still working, is your job Completely Sedentary, Not Completely Sedentary but with Some Rare Physical Activity, Requires Moderate Physical Activity, Requires Vigorous Physical Activity), and leisure activity (What best describes your leisure activity level?; not active at all, rarely active, prefer sedentary activities, moderately active at least three times per week, vigorously active for at least 30 min, three times per week). The data the subject was enrolled in was also recorded and used to determine the meteorological season (Spring: March, April, and May; Summer: June, July, and August; Fall: September, October, and November; Winter: December, January, and February).

### 2.3 ECG Patch Monitoring Protocol

After the restoration of sinus rhythm and upon determining the patient's medical stability, a research staff member applied a 14‐day ECG patch (Zio XT, iRhythm, Chicago, IL) to the chest. The placement of the patch followed the manufacturer's recommendations: it was positioned on the flattest part of the upper left chest, approximately one finger width below the collarbone, centered over the left pectoral muscle. The edge of the Zio XT ECG Patch was aligned next to the sternum, with the arrow on the top pointing upward. Care was taken to avoid the armpit and breast tissue.

One of the main advantages of using the Zio XT ECG Patch is that it imposes minimal activity restrictions, allowing subjects to resume their normal daily activities while wearing the patch, except for swimming. Subjects were instructed to wear the ECG patch for the recommended 14‐day period and not to scratch in or around the patch to minimize artifacts on the ECG recording. Additionally, subjects were informed to promptly notify a research staff member if the ECG patch fell off. In cases where premature removal of the patch occurred, the patient would contact the research staff member to provide the reason. If the patient failed to make contact, the research staff member would contact the patient to ensure documentation of the reason for premature removal. The data regarding premature removal was coded to reflect various categories, including loss of adhesive/ECG patch falling off, adverse reaction to the adhesive, technical issues with the patch, early termination of the study, illness or hospitalization, or refusal to continue participation.

### 2.4 Statistical Analysis

We examined the data's normality by inspecting the distribution curve and the Kolmogorov–Smirnov test. No data was missing. However, the days of ECG recording, AF burden, height, and BMI were not normally distributed. We categorized age and BMI based on quartiles. We defined high compliance as wearing the ECG patch for the recommended duration of 14 days. It is important to note that this is consistent with data published by the manufacturer, which states that 98% of individuals are compliant with 14 days of monitoring.

We describe the sample using descriptive statistics, which include frequencies (%), means (± standard deviation, SD) if the variables are normally distributed, or medians (interquartile range, IQR) if they are not. To assess group differences, we used various tests, including the independent sample *t*‐test and one‐way ANOVA for normally distributed variables, the Mann–Whitney *U* test, the Kruskal–Wallis test for non‐normally distributed variables, and the *χ* ^2^ test for categorical variables.

Afterward, we used logistic regression as a generalized model to identify predictors of compliance. We investigated using a Poisson regression model, but the data was over‐dispersed, making the reported estimates unreliable. Predictors of specific interest include age, sex, race, ethnicity, BMI, work status, job activity, leisure activity, and season. We dichotomized weight and BMI at the median for additional analyses. Statistical significance was set at *p* < 0.05. All data was analyzed in SPSS (Armonk, New York), version 27.0.

## 3 Results

### 3.1 Sample Descriptive

In this study, 214 subjects (67%, *n* = 143 male; 97%, *n* = 208 White; 95%, *n* = 204 non‐Hispanic) were enrolled, with most subjects undergoing an ablation procedure (59%, *n* = 127) while the remaining underwent cardioversion (41%, *n* = 87). Many subjects reported being moderately (44%, *n* = 94) or vigorously active (21%, *n* = 45) and not currently working (58%, *n* = 124). The median BMI was 31.4 (IQR 7.7) kg/m^2^, which indicates that the average patient was obese, according to published guidelines.

**FIGURE 1:**
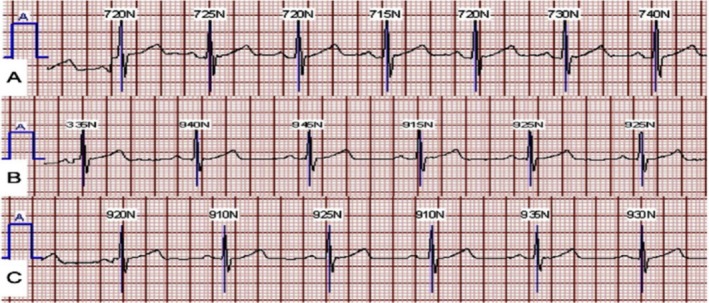
Panels (A), (B), and (C) show a 7‐beat ECG collected from days 1, 7, and 14, respectively, from a 57‐year‐old male (BMI 25 kg/m^2^) who had high compliance with the ECG patch. Given his high compliance, the interpretability of the ECG recordings is maintained as exhibited in the figure.

Compliance with the ECG patch was very high, with a median wear time of 13.2 (1.87) days, and the mean wear time was 11.6 (±3.94) days. Approximately 41% (*n* = 88) of subjects completed all 14 days and were therefore classified as highly compliant. An example of the ECG tracing from the ECG patch over time is seen in Figure 1.

**FIGURE 2:**
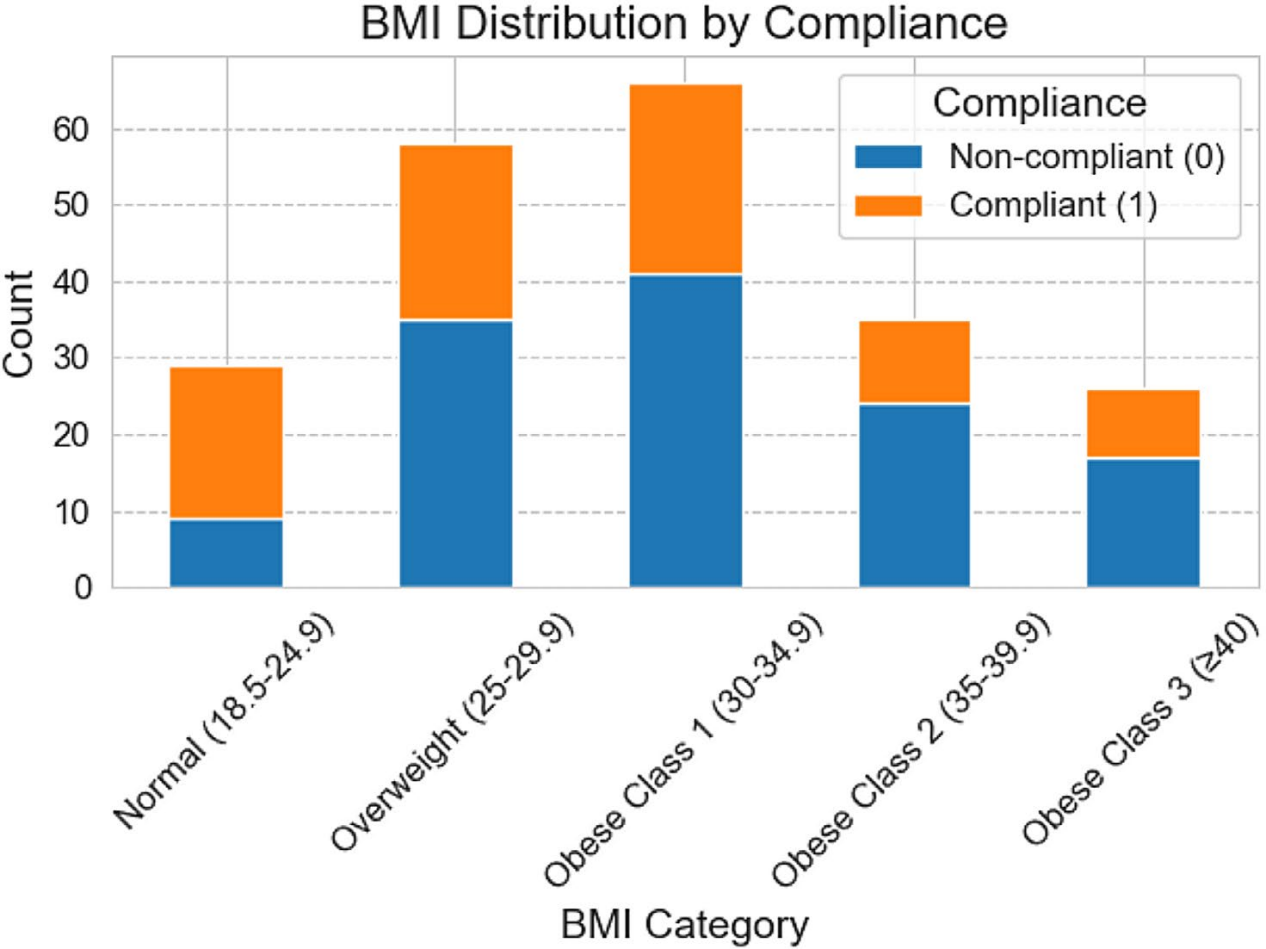
BMI negatively impacts wear time compliance with a 14‐day ECG patch.

Figure 2 illustrates the distribution of compliance across BMI categories. Notably, patients in the higher BMI groups (Obese Class 2 and 3) had lower compliance rates, with a greater proportion of non‐compliant individuals compared to those with normal or overweight BMI. This trend is consistent with our statistical findings, reinforcing the association between higher BMI and premature ECG patch removal. The statistical results can be reviewed in Table 1.

**TABLE 1:** Subject compliance of 14‐day ECG patch based on BMI category.

BMI category	Total *N*	Compliant	Non‐compliant	*p*
Normal	29	20 (69%)	9 (31%)	REF
Overweight	59	23 (39%)	36 (61%)	0.01
Obese class I	67	25 (37%)	42 (63%)	< 0.01
Obese class II	33	11 (33%)	22 (66%)	< 0.01
Obese class III	26	9 (35%)	17 (65%)	0.02
Total	214	88	126	

### 3.2 AF Burden Measured on ECG Patch

Among the subjects enrolled in this study, 31.3% (*n* = 67) experienced AF, as evidenced by the ECG patch. AF incidence (low compliance 34.1%, *n* = 43/126; high compliance 27.2%, *n* = 24/88, *p* = 0.29) nor AF burden (low compliance 0.12 ± 0.26; high compliance 0.08 ± 0.22, *p* = 0.28) differed between those subjects with high and low compliance with the ECG patch. Similarly, the likelihood of measuring a long AF episode (26%, *n* = 55) was not more likely to be captured among subjects with high compliance with the ECG patch compared to those with low compliance (low compliance 30.0%, *n* = 34/126; high compliance 23.9%, *n* = 21/88, *p* = 0.61). There was no difference in AF incidence between patients who underwent cardioversion (33%, *n* = 29/87) or ablation (29.9%, *n* = 38/127; *p* = 0.60). Interestingly, the 14‐day ECG patch recording duration in minutes and AF burden were poorly correlated (*ρ* = −0.03, 95% CI −0.161− 0.115 *p* = 0.73).

### 3.3 Reasons for Poor Compliance

Among the 126 subjects with poor compliance (59%, *n* = 126/214), the most commonly cited reasons were: failure to comply with protocol (59%, *n* = 74); a loss of adhesive (22%, *n* = 27); subject refusal to continue to participate in the study (7%, *n* = 9); technical issues with study equipment (7%, *n* = 9); hospitalization (3%, *n* = 4); and reaction to adhesive (2%, *n* = 3). Note that we specifically excluded patients with known allergies to adhesives. Subjects with higher weight (2.7% *n* = 3/111; 17.5% *n* = 18/103, *p* = 0.01) and BMI (5.6% *n* = 6/107; 14.0% 15/107, *p* = 0.04) were more likely to have issues with adhesive and the patch falling off prematurely. This data can be reviewed in Table 2.

**TABLE 2:** Comparison of BMI categories across adhesive issues.

BMI category	Total *N*	Fell off/adhesive	*p*
Normal	29	1 (3%)	REF
Overweight	59	3 (5%)	0.66
Obese Class I	67	9 (13%)	0.14
Obese Class II	33	8 (24%)	0.02
Obese Class III	26	6 (23%)	0.03
Total	214	27	

### 3.4 Patient Characteristics and Between Good and Poor Compliance and Predictors of Poor Compliance

Subject characteristics were compared between patients exhibiting good and poor compliance with the ECG patch (Table 3). Subjects who exhibited good compliance were comparable to subjects that exhibited poor compliance, except that subjects with poor compliance tended to have a greater weight (99.8 kg (30.8); 92.1 kg (24.9), *p* = 0.03) and had a higher BMI (31.89 kg/m^2^(8.50); 30.50 kg/m^2^ (8.03), *p* = 0.01). Wear time was nearly 6 h less on average among the subjects with the highest BMIs compared to the subjects with normal BMIs (320.52 (39.38); 314.85 (48.33) *p* = 0.05) and about 5 h less among the subjects with the highest weights (320.95 (34.6); 315.27 (53.63), *p* = 0.03).

**TABLE 3:** Subject characteristics overall and in‐comparison with ECG patch compliance, *n* = 214.

Characteristic	Mean (±standard deviation)/median (interquartile range)/% (*n*)	Subject with poor compliance (59%, *n* = 126)	Subject with high compliance (41%, *n* = 88)	*p*
Age (years)	65 (+9.5)	65 (+9.5)	65 (±9.5)	0.75
% Male	67% (143)	67% (85)	66% (58)	0.81
% White race	97% (208)	96% (121)	99% (87)	0.22
% Non‐Hispanic	95% (204)	96% (121)	94% (83)	0.56
% Smoking history	44% (94)	48% (60)	39% (34)	0.19
% Ablation	59% (127)	56% (71)	64% (56)	0.29
% Atrial fibrillation after procedure	31% (67)	34% (43)	27% (24)	0.29
Weight (kg)^a^	**97.98 (63)**.	**99.79 (30.8)**	**92.07 (24.9)**	**0.03**
Height (m)^a^	70 (5)	70 (5)	70 (6)	0.88
Body mass index (kg/m^2^)^a^	**31.4 (7.72)**	**31.89 (8.50)**	**30.50 (8.03)**	**0.01**
Total ECG recording (days)	**317.3 (45.0)**	**302.88 (161.91)**	**336.02 (2.96)**	**< 0.01**
AF burden^a^	10.2% (±24%)	11.8% (±25.5)	7.89% (±22.1)	0.28
Work status				
Not currently working	57.9% (124)	57.9% (73)	58.0% (51)	0.18
Part time	9.8% (21)	12.7% (16)	5.7% (5)	
Full time	32.2% (69)	29.4% (37)	36.4% (32)	
Job activity (*n* = 90)		*N* = 53	*N* = 37	
Completely sedentary	12.2% (11)	13.2% (7)	10.8% (4)	0.53
Not completely sedentary but with rare physical activity	52.2% (47)	49.1% (26)	57% (21)	
Moderate physical activity	25.6% (23)	30.2% (16)	18.9% (7)	
Vigorous physical activity	10% (9)	9.4% (4)	13.5% (5)	
Leisure activity				
Not active at all	3% (6)	1% (1)	5.6% (5)	0.16
Rarely active	31.8% (68)	34.1% (43)	28.4% (25)	
Moderately active	44.1% (94)	44.4% (56)	40.4% (38)	
Vigorously active	21.0% (45)	20% (25)	22.7% (20)	
Season				
Spring (March–May)	26.9% (57)	27% (34)	26.1% (23)	0.41
Summer (June–August)	31.3% (67)	34.9% (44)	26.1% (23)	
Fall (September–November)	27.6% (59)	26.2% (33)	29.5% (26)	
Winter (December–February)	14.5% (31)	11.9% (15)	18.2% (16)	

^a^ Days of recording, weight, height, BMI, and AF burden were not normally distributed; median (IQR) reported.Note: Significance of underlined value indicates statistical significance.

Using a logistic regression model, we noted that BMI (*B* = −0.046, OR = 0.955 95% CI 0.916–0.996 *p* = 0.033) and weight (*B* = −0.007, OR‐0.0993 95% CI 0.988–0.999 *p* = 0.028) were a statistically significant independent predictors of poorer compliance with the ECG patch. Full‐time or part‐time employment and moderate or vigorous activity were not predictors of poor compliance (*p* > 0.05).

## 4 Discussion

Our study investigated factors affecting ECG patch monitoring duration and quality in 214 patients with AF undergoing catheter ablation or cardioversion. We found that only 41% of patients achieved the full 14‐day monitoring period, with a median wear time of 13.2 days. Notably, BMI emerged as an independent predictor of poorer compliance, with higher BMI associated with shorter wear times. The most common reason for premature device removal was loss of adhesive (22%), particularly among patients with higher BMI.

The challenges in diagnosing AF are well‐documented. The EORP‐AF Pilot General Registry reported that 39.7% of patients were asymptomatic, and among symptomatic patients, 51.2% had only mild and non‐specific symptoms (Boriani et al. 2015). Furthermore, the RealiseAF Study demonstrated that among 9816 patients, 50.3% had either paroxysmal (26.5%) or persistent AF (23.8%) (Chiang et al. 2012). This unpredictable nature of AF, combined with limited symptomatology, necessitates extended monitoring periods for accurate diagnosis and management. Consequently AF burden is becoming a more popular measurement of AF severity (Varma et al. 2021; January et al. 2019).

Traditional monitoring approaches using Holter monitors have shown limited effectiveness to newer patchbased devices. The 24 h ECG Holter detects new AF in only 2%–3% of patients (Nagata et al. 2020), whereas ECG patch devices, recording continuously for up to 14 days, demonstrate significantly higher detection rates (Rosero et al. 2013; Varma et al. 2021). These extended wear devices improve AF detection and provide crucial insights into AF burden and underlying atrial remodeling processes (Chua et al. 2020; Cheung et al. 2014). Liu et al. (2021) reported that among 158 patients using 14‐day ECG patches (mean wear time: 12.3 ± 3.2 days), arrhythmia detection rates significantly exceeded those of traditional 24‐h Holter monitoring, findings replicated by Barrett et al. (2014) and others.

However, real‐world compliance with ECG patches appears to be lower than manufacturer‐reported rates. A scoping review of wearable ECG devices found that the median wear time for ECG patches is approximately 11–12 days (Bouzid et al. 2022), aligning closely with our findings. This discrepancy suggests that patient‐specific factors, such as BMI, may influence adherence and necessitate tailored monitoring strategies.

Our study makes several novel contributions to the field. First, we identified BMI as a significant predictor of monitoring compliance. This finding is particularly relevant because obesity is not only associated with a 50% increased incidence of AF but is also the second largest attributable risk factor after hypertension. Physiologically, obesity increases low‐grade systemic inflammation, driving atrial remodeling and fibrosis (Fraley et al. 2005; Dzikowicz and Carey 2019). Thus, patients who may benefit most from longer‐term monitoring are those with a higher BMI. Second, we found an unexpected lack of relationship between AF burden and monitoring duration, contrary to the hypothesis that longer monitoring would capture more AF episodes. Early recurrence of AF after cardioversion and ablation typically ranges between 20% and 40% in the first 2 weeks (Darby 2016), and our sample's recurrence rate of 31% aligns with these estimates. However, the AF burden did not significantly differ between high and low compliance.

Furthermore, while previous research has suggested that ECG signal quality remains stable over time, these studies were conducted in small populations with low BMI (Campero Jurado et al. 2023). Our study highlights the need for further investigation into whether higher BMI influences signal degradation, particularly due to increased skin impedance, motion artifacts, and adhesive failure in overweight patients.With that said newer contactless modalities may be more benefical as they overcome this potential problem (Couderc et al. 2015; Rosenberg et al. 2013).

Several limitations should be considered when interpreting our results. First, our study population was predominantly White (97%) and non‐Hispanic (95%), potentially limiting generalizability. Second, we only evaluated one type of ECG patch device; different devices might show varying levels of adherence and compliance. Third, our study focused on post‐procedure patients, and findings might differ in treatment‐naive patients or those with different AF patterns.

Our findings have important implications for clinical practice and device development as the ECG patch market continues to grow significantly in the US (compound annual growth rate of 19.58% 2023‐2030). Clinicians may need to consider BMI when selecting monitoring strategies to ensure that the monitor selection they choose is suitable for their patients and ensures the capture of high‐quality data. Our results highlight device manufacturers' need for improved adhesive technologies and designs that better accommodate patients with higher BMIs. Further development and testing are necessary to ensure these patches can effectively adhere to the skin across diverse patient populations.

The findings from this analysis contribute to the growing body of evidence supporting personalized approaches to AF monitoring. Understanding these factors affecting monitoring compliance becomes increasingly essential for optimizing patient care and improving AF detection and management strategies, particularly in high‐risk populations.

## 5 Conclusions

In conclusion, we determine that a large sample of patients undergoing intervention for AF is highly compliant with a 14‐day ECG patch. Current or former smoking and BMI were predictors of low compliance. While ECG patches are becoming increasingly popular, further research on compliance and the moderating effects of obesity is warranted.

## Author Contributions

D.J.D.: conceptualization, Duration curation, formal analysis, methodology, supervision, Writing – original draft. M.A.: formal analysis, resources, software, visualization, Writing – review and editing. B.M.: data curation, project administration, resources. X.X.: resources, software. W.Z.: resources, supervision. J.P.C.: funding acquisition, investigation, methodology, project administration, resources, writing – review and editing.

## Conflicts of Interest

Wojciech Zareba is an Editorial Board member of Annals of Noninvasive Electrocardiology and a co‐author of this article. To minimize bias, they were excluded from all editorial decision‐making related to the acceptance of this article for publication.

## Data Availability

The data may be shared upon reasonable request to the PI of the study (Couderc).
